# NMR Reaction Monitoring Robust to Spectral Distortions

**DOI:** 10.1021/acs.analchem.5c00800

**Published:** 2025-07-16

**Authors:** Barbara Domżał, Magdalena Grochowska-Tatarczak, Przemysław Malinowski, Błażej Miasojedow, Krzysztof Kazimierczuk, Anna Gambin

**Affiliations:** † Faculty of Mathematics, Informatics and Mechanics, 49605 University of Warsaw, Banacha 2, Warsaw 02-097, Poland; ‡ Centre of New Technologies, 49605 University of Warsaw, Banacha 2C, Warsaw 02-097, Poland

## Abstract

Nuclear magnetic resonance spectroscopy (NMR) is one of the most potent analytical chemistry methods, providing unique insight into molecular structures. Its noninvasiveness makes it a perfect tool for monitoring chemical reactions and determining their products and kinetics. Typically, the reactions are monitored by a series of ^1^H NMR spectra acquired at regular time intervals. Even such a straightforward approach, however, often suffers from several problems. In particular, the reaction may cause sample inhomogeneity, resulting in a nonhomogenous magnetic field and distorted spectral lineshapes. When the studied process is fast, hardware correction (shimming and locking) cannot be applied on the fly, and the spectral quality degrades over the course of the reaction. Moreover, when nondeuterated solvents have to be used in the reaction mixture, a magnetic field-stabilizing system (deuterium lock) cannot work. Consequently, the spectra have distorted lineshapes, reduced resolution, and randomly varying peak positions, making them challenging to analyze quantitatively with standard software. In this paper, we propose a conceptually new approach to the quantitative analysis of a series of distorted spectra. The method is based on the Wasserstein distance and can effectively quantify the components of a reaction mixture without the need for peak-picking. We provide open-source software requiring minimum input from the user, i.e., a set of spectra indexed by time.

## Graphical abstract

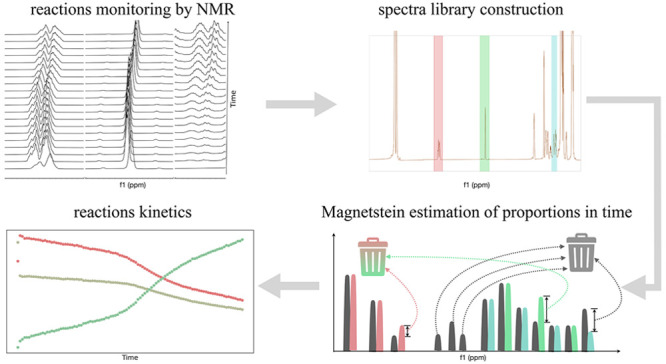

NMR spectroscopy is widely used in chemical analysis. It is noninvasive, requires minimal sample preparation, and has increasingly better detection limits. High-resolution NMR spectrometers, based on superconducting magnets, can currently measure compounds at sub-μM concentrations. Another advantage of NMR is that it provides detailed information about molecular structures and their changes. Thus, NMR is ideally suited to the monitoring of chemical reactions.^1–3^

In the most straightforward approach, reactions are carried out in a standard NMR tube and monitored using spectra recorded at regular time intervals.^4–6^ In a more advanced approach, the reaction is carried out in an external reactor, and the reaction mixture is pumped through an NMR probe.^3,7,8^ The experiment typically involves a series of one-dimensional (1D) ^1^H NMR spectra, since they are fast to collect. The techniques can be improved by the application of advanced pure-shift methods^9^ or dedicated data processing.^10^ The two-dimensional (2D) methods accelerated by the use of nonuniform sampling^11–13^ or single-scan techniques^14,15^ have also gained growing attention.

The perfect 1D NMR signal is a sum of exponentially decaying oscillations, and its spectrum is a sum of Lorentzian “peaks” whose positions in the frequency domain and integrals are related to the molecular structure. To analyze the kinetics of a process monitored using serial NMR measurement, one has to find peaks in each of the collected spectra and integrate them. This procedure, known as peak-picking, is more complex than it may seem, as the ^1^H NMR spectra, although sensitive and fast to measure, often suffer from severe peak overlap. The peak-picking programs must employ the most advanced methods, such as machine-learning,^16–18^ to decompose an NMR spectrum into a set of peaks and determine their parameters. Alternative approaches, such as CRAFT, can determine the parameters directly from a time-domain signal.^19,20^ Notably, these approaches are effective only if the right model of a peak shape is assumed (e.g., Lorentzian or Gaussian function). However, various measurement imperfections can distort the shape of the spectral lines and make them far from ideal.

Magnetic field disturbances are typical measurement imperfections in NMR experiments and can strongly affect the spectral quality. In routine measurements, the magnetic field is typically stabilized using a deuterium “lock”^21^ sometimes supported by additional systems.^22^ The spatial homogeneity of a magnetic field within the receiver coil is ensured by setting appropriate currents in the correction coils, i.e., by “shimming”. However, both “locking” and “shimming” can be difficult or even impossible in the case of reaction monitoring. The former usually requires deuterated solvents, which may affect the studied reaction. The latter is time-consuming and thus cannot be performed if the studied reaction is fast. For the reaction monitoring performed in a flow mode, the imperfections can be even more pronounced.^23^ The line shape distortions are often corrected using a reference deconvolution method.^24^ However, the method requires the spectroscopist to guess the parameters, amplitude, half-width, and frequency, of one, well-resolved peak. This is often problematic. Also, the method is cumbersome when applied to the processing of massive data sets, common in reaction monitoring, since the reference selection has to be repeated for each spectrum.

In this paper, we propose a method of spectral analysis that is suited for the difficult cases of NMR reaction monitoring since it is robust to line shape imperfections. It does not assume any specific line shape functions, so the distortions caused by lack of shimming or locking are less problematic than in conventional approaches. The peak overlap is also better resolved. The method is based on the Wasserstein distance,^25^ which is a mathematical notion taken from the optimal transport theory. It has been previously shown^26–29^ that the specific properties of this metric make it a suitable tool for analysis of difficult spectroscopic data. The Wasserstein distance can be calculated between any two spectra, and no assumptions about the shapes of peaks are needed. Moreover, the metric is robust to overlap of signals from different reagents and small changes in position along the chemical shift axis. Our method treats the problem of estimating relative amounts of components changing over time as the problem of regression with the Wasserstein distance. As a result, we obtain a sequence of proportions in consecutive time points, allowing us to infer the kinetics of the monitored reaction.

## Theoretical Background

The set of L chemical reactions between *k* substances (*reagents*) *R*_1_, *R*_2_, ..., *R*_*k*_ can be described by the following general scheme:

$${\mathrm{v̲}}_{l1}R_{1}+{\mathrm{v̲}}_{l2}R_{2}+\cdot \cdot \cdot +{\mathrm{v̲}}_{lk}R_{k}\to {\mathrm{v̅}}_{l1}R_{1}+{\mathrm{v̅}}_{l2}R_{2}+\cdot \cdot \cdot +{\mathrm{v̅}}_{lk}R_{k}$$

where ${\mathrm{v̲}}_{lj}$ and ${\mathrm{v̅}}_{lj}$ are the amounts of *j*-th substrate and *j*-th product, respectively, in *l*-th reaction, *l* = 1, 2, ..., *L*. We assume that for every *l* = 1, 2, ..., *L* and every *j* = 1, 2, ..., *k* either ${\mathrm{v̲}}_{lj}$ or ${\mathrm{v̅}}_{lj}$ is equal to 0 (in other words, substance *R*_*j*_ is involved in *l*-th reaction either as substrate or as product).

To simplify the notation, we can add up all the L reaction equations to create the following one:

$$\left(\overset{L}{\underset{l=1}{\sum }}{\mathrm{v̲}}_{l1}\right)R_{1}+\left(\overset{L}{\underset{l=1}{\sum }}{\mathrm{v̲}}_{l2}\right)R_{2}+\cdot \cdot \cdot +\left(\overset{L}{\underset{l=1}{\sum }}{\mathrm{v̲}}_{lk}\right)R_{k}\to \left(\overset{L}{\underset{l=1}{\sum }}{\mathrm{v̅}}_{l1}\right)R_{1}+\left(\overset{L}{\underset{l=1}{\sum }}{\mathrm{v̅}}_{l2}\right)R_{2}+\cdot \cdot \cdot +\left(\overset{L}{\underset{l=1}{\sum }}{\mathrm{v̅}}_{lk}\right)R_{k}$$

and defining

$${\mathrm{v̲}}_{j}=\overset{L}{\underset{l=1}{\sum }}{\mathrm{v̲}}_{lj},,{\mathrm{v̅}}_{j}=\overset{L}{\underset{l=1}{\sum }}{\mathrm{v̅}}_{lj}$$

we arrive at

1 $${\mathrm{v̲}}_{1}R_{1}+{\mathrm{v̲}}_{2}R_{2}+\cdot \cdot \cdot +{\mathrm{v̲}}_{k}R_{k}\to {\mathrm{v̅}}_{1}R_{1}+{\mathrm{v̅}}_{2}R_{2}+\cdot \cdot \cdot +{\mathrm{v̅}}_{k}R_{k}$$

We are going to use eq 1 to describe all the substrates and products that are involved in any of *L* chemical reactions, and their amounts.

Now, let us introduce the state *p*_*t*_ of the system described by eq 1 in moment *t*. Let [*R*_*j*_]­(*t*) denote the concentration of reagent *R*_*j*_ in moment *t*. We are going to encode the state as the vector of concentrations of the reagents *R*_1_, *R*_2_, ..., *R*_*k*_ involved in (1) in the moment of time *t*, normalized by the sum of all the concentrations, i.e.:

2 $$\begin{array}{ll} p_{t} & =\left(p_{1,t},p_{2,t},...,p_{k,t}\right),,where\\ p_{j,t} & =\frac{\left[R_{j}\right]}{\overset{k}{\underset{i=1}{\sum }}\left[R_{i}\right]\left(t\right)},\mathrm{for}\;j=1,2,...k \end{array}$$

The aim of our analysis is to estimate *p*_*j*,*t*_ for all *j*, *t* to be able to infer the reactions’ kinetics. We assume that our data set consists of *T* spectra μ_*t*_ of the reaction mixture measured in consecutive moments *t* = 1, 2, ...*T*. In principle, each of the time points *t* can be analyzed separately and independently, and, for the sake of clarity, we will describe the working of our method on the single reaction mixture spectrum in the fixed moment *t*. However, later on, we will show that the results obtained for preceding moments can be used to accelerate the computations.

To perform estimation, first, we need to construct a *library*, that is, a set of spectra representing individual reagents. An element of the library does not need to be a full spectrum of the reagent; it can be a spectrum cut down to regions or even one area involving a single peak. For instance, such an object can be obtained by choosing chemical shift intervals corresponding to substrates from the very first spectrum, measured when there is still a considerable amount of substrates in the reaction mixture. Similarly, for the product, it can be retrieved from the spectrum measured for *t* = *T*. An alternative approach would be to provide the spectra of reagents measured separately outside the reaction mixture, as in our previous work.^27^

The core of our method is the Magnetstein algorithm.^27^ We summarize its main ideas briefly in the Supporting Information.

The algorithm is implemented in the Python package.^27^ The problem of computing p_j,t_ is formulated as regression that boils down to a linear program and, as already mentioned, can be solved separately and independently for every fixed moment *t*. However, at this point, we make use of the knowledge that spectra μ_*t*–1_ and μ_*t*_, being close in time, are expected to be similar to one another. Linear programs are solved using the simplex algorithm, which can be *warm-started*, i.e. begin the search for the optimal solution from some predefined proposal values, and this is where we use the information from the previous time point, see Supporting Information for details.

An important point to note here is that, although the Magnetstein’s purpose is to estimate the values (2), the output of the algorithm *p*_1,*t*_, *p*_2,*t*_, ..., *p*_*k*,*t*_ does not necessarily sum up to 1 due to the possible presence of noise and contamination in the data. The value of expression *p*_0,*t*_ ≔ 1 – *p*_1,*t*_ – *p*_2,*t*_ – ··· – *p*_*k*,*t*_ is Magnetstein’s estimation of the proportion of contamination in the mixture’s spectrum. By introducing this additional way of dealing with excessive signal in the mixture’s spectrum, we make the algorithm robust to an incomplete library. Namely, suppose that the user did not provide all the spectra of the reagents as input to the algorithm. This will not perturb the shape of the resulting kinetic curves, provided that the program’s parameters were reasonably tuned. Indeed, the signal corresponding to the component missing from the library will be deemed noise. In some favorable cases, by analyzing the graph of *p*_0,*t*_ against time, one can even reconstruct the behavior of the missing reagent, see Supporting Information for details. By introducing the quantity *p*_0,*t*_, we also ensure the equivalence between the concentration- and proportion-based data representation.

## Methods

To test the method, we chose three reactions with a gradually increasing degree of complexity of spectral analysis. First, we monitored the easiest reaction, sucrose hydrolysis, providing spectra with only slightly broadened and well-resolved peaks. Then, we monitored two Lewis-acid-catalyzed hydrosilylation reactions in nondeuterated solvents in which a lock system could not be used.^30^ Moreover, the fast kinetics of those processes made it impossible to perform gradient shimming before acquisition of the first spectrum. In consequence, the resulting spectra contained shifting peaks with distorted lineshapes. Additionally, one of the hydrosilylation reactions provided spectra with severe peak overlap, hampering conventional analysis.

As the analyzed data were collected during an ongoing reaction, it was impossible to obtain a ground-truth amount of each reagent in the given moment of time. Thus, the natural way to assess the accuracy of Magnetstein’s output was to perform analogical analysis using a well-established tool (in this case, Mnova software) and compare the results against each other. Note that the general correctness of Magnetstein’s estimation has already been proven on other examples where the ground truth was known.^27^

### Sample Preparation and Reaction Monitoring

The first reaction, enzymatic hydrolysis of sucrose, was performed in a standard 5 mm NMR tube directly on a spectrometer magnet. It proceeded with an immediate follow-up reaction of α-glucose conversion to β-glucose. The sucrose hydrolysis has been performed as described previously.^12^ Namely, we mixed stock solutions of 2.0 M sucrose in D_2_O and the enzyme in a 1:1 volumetric ratio. We obtained the enzyme’s stock solution by dissolving β-D-fructofuranosidase (Sigma-Aldrich) in an acetate buffer (Sigma-Aldrich) (95 mM, H_2_O, pH = 5.2) to reach the concentration of 4.6 μg/mL.

Hydrosilylation reactions were prepared by adding a catalyst solution to a silane/olefin mixture in 1,2-difluorobenzene, added in a 1:1 ratio. Triethylsilane (ThermoScientific) was used as received. 1-hexene (Sigma-Aldrich) and 2-pentene (Sigma-Aldrich, cis- and trans-mixture) were dried over CaSO_4_. 1,2-difluorobenzene (Fluorochem) was distilled over P_2_O_5_. The catalyst used was a pseudobinary calcium salt of perfluoro­(tertbutoxy)­aluminate anion (Ca­[Al­(OC­(CF_3_)_3_)_4_]_2_, further denoted as Ca­[pf]_2_), obtained in a metathesis reaction, according to the synthetic protocol reported in the literature:^30^

$${CaCl}_{2}+2Ag\left[Al{\left({OC\left({CF}_{3}\right)}_{3}\right)}_{4}\right]\overset{{\mathrm{SO}}_{2}}{\to }Ca{\left[Al{\left({OC\left({CF}_{3}\right)}_{3}\right)}_{4}\right]}_{2}+2AgCl↓$$

CaCl_2_ was dried in a furnace at 200^◦^C, and immediately transferred afterward into the glovebox with ca. 30 min treatment of dynamic vacuum in an antechamber, to remove traces of moisture from the chloride. Ag­[pf] was synthesized according to the literature procedure.^31^ Liquid SO_2_ (Air Liquide) was dried over CaH_2_. Due to the instability of the [pf]^−^ anion in the presence of water, all the manipulations were performed in the glovebox in the argon atmosphere, in anhydrous and anaerobic (O_2_ < 2 ppm) conditions. Liquid SO_2_ was condensed on a Schlenk line. The spectra were measured in a 5 mm NMR tube sealed with a septum. The substrates and catalyst solutions were prepared in the glovebox in glass flasks sealed with septa. The syringes used to handle the solutions were rinsed with acetone before use to remove any grease from their surface. All glassware, including NMR tubes, was dried before use by treatment of vacuum when heated to ca. 100–150 °C.

For a hydrosilylation reaction with 2-pentene, a concentrated (8.7 M) substrate solution was prepared by adding 1.7 M 2-pentene and triethylsilane to 0.2 mL of 1,2-difluorobenzene to a 5 mm NMR tube sealed with a septum. The catalyst solution was prepared by adding 0.02 mmol (37 mg) of Ca­[pf]_2_ to 0.3 mL of 1,2-difluorobenzene The substrate mixture was measured first, then the catalyst solution was added dropwise, with a syringe. All spectra were recorded at a slightly elevated (35 °C) temperature.

For a hydrosilylation reaction with 1-hexene, a substrate mixture consisting of 0.86 mmol of both 1-hexene and triethylsilane was prepared in 1,2-difluorobenzene in a 5 mm NMR tube sealed with a septum. The catalyst solution was prepared by adding 0.026 mmol (51 mg) of Ca­[pf]_2_ to 1,2-difluorobenzene. Similarly, as in the case of 2-pentene, the substrate mixture was measured first, and then the catalyst solution was added dropwise, with a syringe. Due to the high reaction rate, all of the spectra were recorded at 5 °C.

All experiments were carried out at 700 MHz Agilent DirectDrive2 spectrometer with 2 s interscan delay, 45° pulse angle, 2.93 s acquisition time, and one transient per spectrum. The hydrosilylations and sucrose hydrolysis were monitored using 1000 and 1024 spectra, respectively. Gradient shimming, tuning, locking, and pulse calibration were performed for the sucrose sample. For hydrosilylations, the procedures were performed on the “static” (postreaction) sample from one of the earlier experiments performed in similar conditions.

### Conventional Data Processing

The spectra were processed with Mnova software (Mnova 15.0, Mestrelab Research, S. L., Spain). The automatic phasing and baseline correction using Whittaker smoother^32^ were applied. The zero-filling to 128k points and no apodization were used. The manually selected spectral regions were integrated in Mnova software using the “Sum” method (see Supporting Information for details). The results were compared with Magnetstein processing (see below).

### Data Processing Using the Wasserstein Distance

For all of the experiments described, the library was constructed by cutting spectra to the same regions as those manually integrated using Mnova software (see Supporting Information for details). The parameters in Magnetstein were set to κ_mixture_ = κ_components_ = 0.5 for all three reactions. The full workflow is illustrated in Figure 1, and the detailed instructions for the users, ensuring reproducibility, are available in Supporting Information.

**Figure 1:**
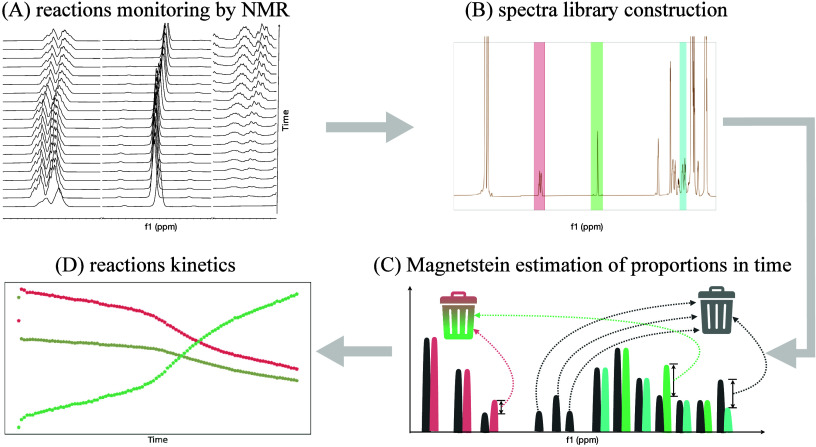
Control flow of data processing. (A) The consecutive NMR spectra are measured during the chemical reaction. (B) One of the measured spectra containing peaks both from substrates and from products is chosen to construct a *library*. The regions corresponding to individual reagents are cut out from the chosen spectrum. The cut fragments constitute the library. (C) The reaction mixture spectra and the library are passed as inputs to the Magnetstein algorithm. The excessive signal from the reaction mixture spectrum and from the library is removed. The estimation of proportions is performed for every time point. (D) The output from the Magnetstein is used to visualize the reaction’s kinetics.

## Results and Discussion

The NMR monitoring of sucrose hydrolysis has been reported previously.^12,33,34^ As shown in Figure 2(A), the hydrolysis leads to fructose and α-glucose, eventually converting to β-glucose. The peaks from the substrate and all three products are well resolved and can be easily integrated by using the conventional approach. Thus, the reaction was used to prove that Magnetstein processing provides results comparable to those of the classical method. The spectra of reaction mixture were cut down to regions as marked in Figure 2(A). Although the parameters in Magnetstein were arbitrarily set to κ_mixture_ = κ_components_ = 0.5, the wide range of settings gave virtually the same results (see Figure 5). Figure 2(B) shows the normalized integrals of selected peaks from compounds involved in the reactions. As can be seen, the general kinetic curves obtained from Magnetstein processing match very well with those from classical integration.

**Figure 2:**
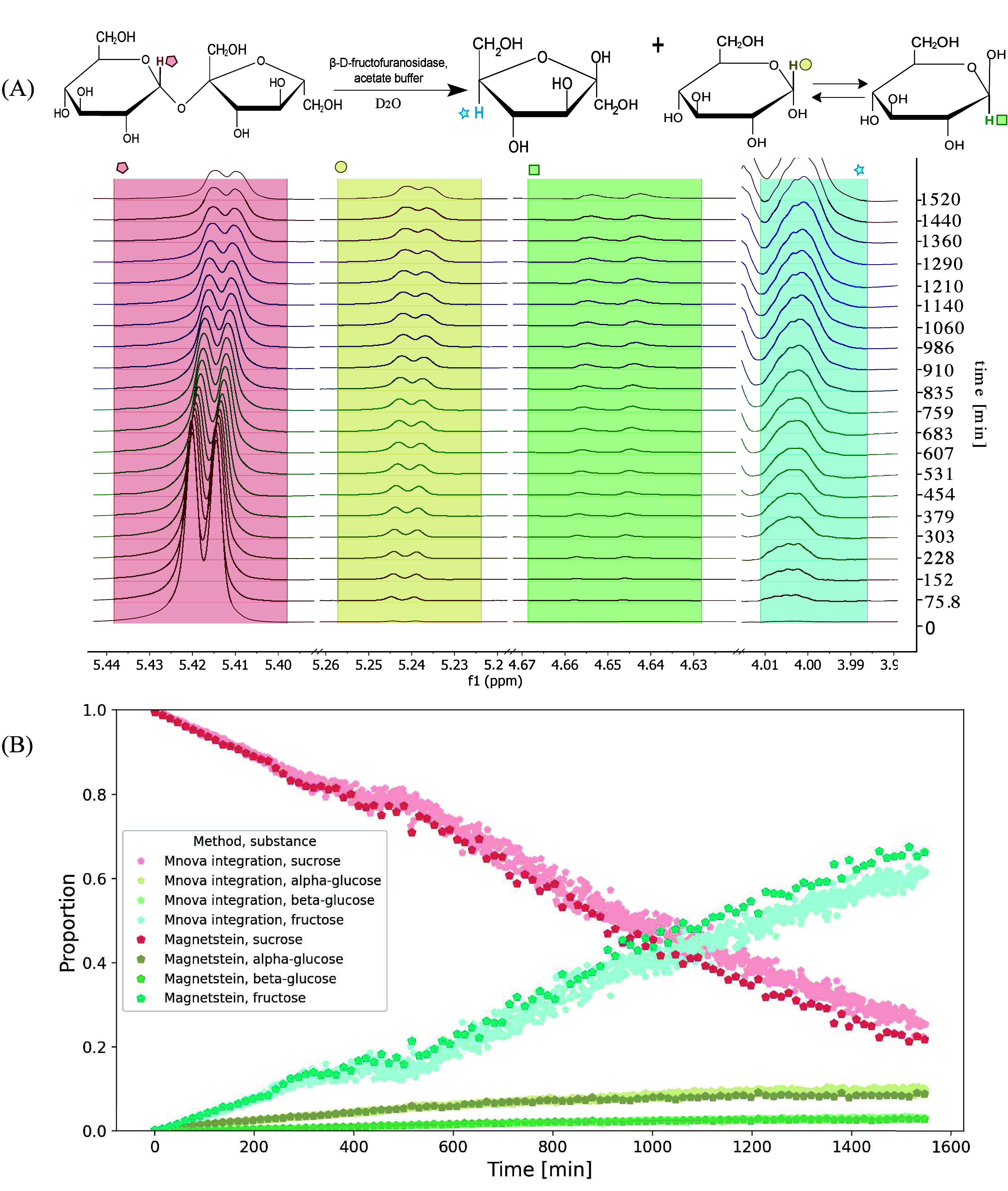
(A) Hydrolysis of sucrose with an immediate follow-up conversion of α-glucose to β-glucose, with every 50th spectrum shown. The same colors and symbols mark the spectral peaks and the corresponding nuclei in the reaction scheme. (B) The kinetics of sucrose hydrolysis. The results of Magnetstein processing (with κ_mixture_ = κ_components_ = 0.5) compared with classical integration in Mnova 15.0.1 program. Colors correspond to regions marked in panel (A).

To test the method for more demanding cases, two Lewis-acid-catalyzed hydrosilylation reactions were monitored^30^ (see Figure 3 and Figure 4). Similarly as for sucrose hydrolysis, the spectra of reaction mixtures were cut down to regions, as marked in Figures 3(A) and 4(A). The reaction and measurement were started immediately after the catalyst was added. However, the addition was performed outside the magnet, and thus, the first several spectra have distorted intensities due to insufficient spin polarization. In addition, they are affected by progressive saturation, since the longitudinal relaxation times of excited nuclei were ca. 10–12 s, while a 45° pulse was used with an interpulse delay of 4.93 s. As mentioned above, there was also no time for proper shimming, and magnetic field stabilization (locking) was impossible due to the lack of deuterated solvent. Thus, in all spectra lineshapes were distorted and deviated far from the usual Lorentzian/Gaussian functions. Due to a lack of a well-resolved singlet, our attempts to perform the reference deconvolution in the Mnova program failed. Still, the peaks in the spectra of 2-pentene hydrosilylation were sufficiently resolved, to allow the simple integration (region-wise summation), whose results match very well with the Magnetstein output (see Figure 3(A). The more difficult case was the hydrosilylation of 1-hexene, which provided spectra with a complete overlap of product and substrate peaks. As shown in Figure 4(B), the conventional integration gives the results differing from the Magnetstein output. Namely, the overlap of integrated product and substrate peaks leads to flattening of sigmoidal kinetic curves (one can imagine that in the extreme case of a full overlap, the kinetic curve would be a constant function). The Magnetstein tool, however, has demonstrated robustness to such peak overlaps in previous studies. This advantage stems from its reliance on optimal transport, which provides a mathematical framework that treats the redistribution of spectral intensity as a flow between defined regions, analogous to redistributing a resource efficiently from one area to another. By jointly analyzing the spectral regions as part of a global optimization, Magnetstein allows for the “movement” of spectral intensity across overlapping peaks in a way that respects the overall conservation of intensity.

**Figure 3:**
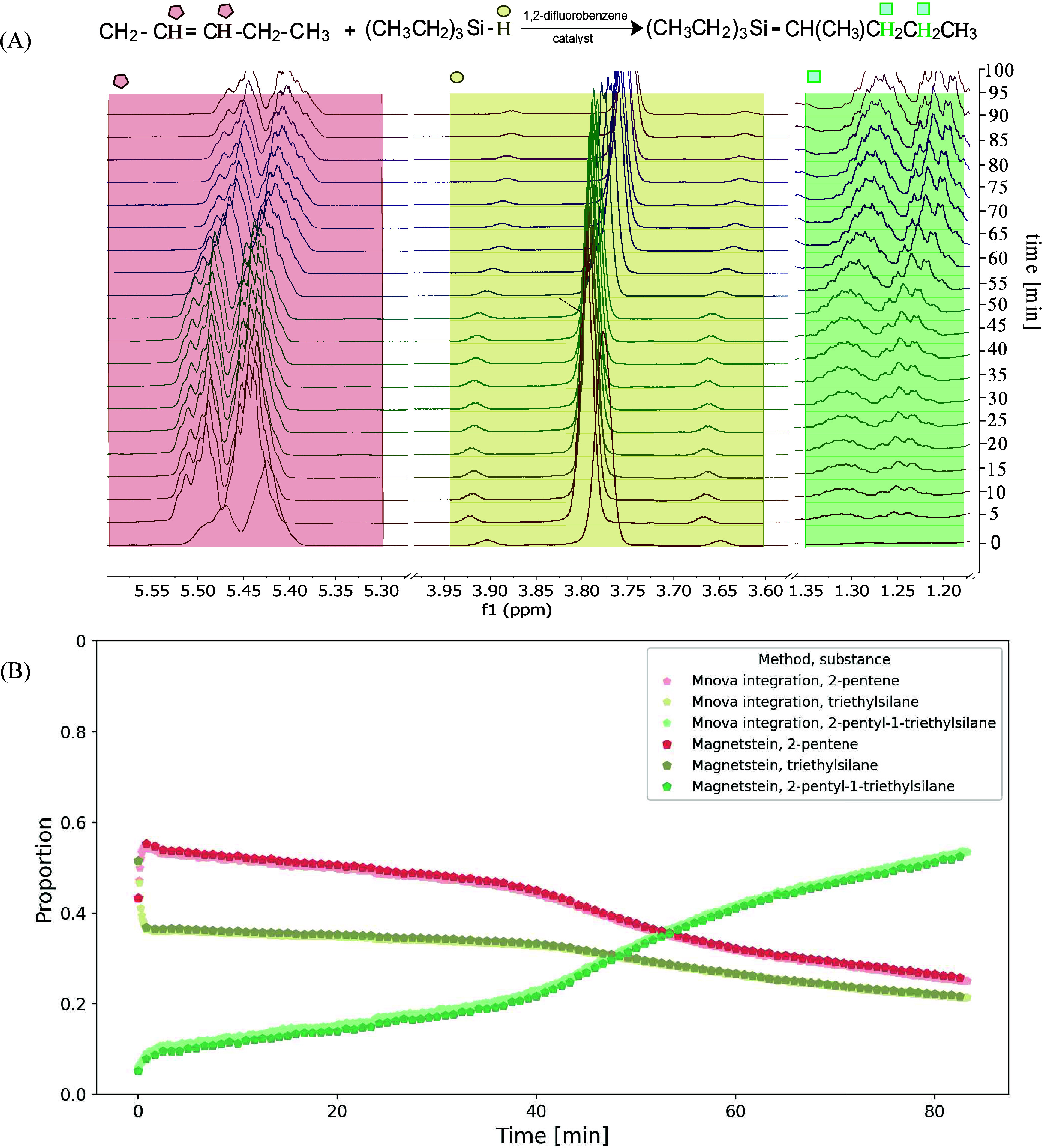
(A) Hydrosilylation reaction of 2-pentene and triethylsilane in 1,2-difluorobenzene, with ∼1.2 m% of a catalyst, with every 50th spectrum shown. The same colors and symbols mark the spectral peaks and the corresponding nuclei in the reaction scheme. (B) The kinetics of pentene hydrosilylation. The results of Magnetstein processing (with κ_mixture_ = κ_components_ = 0.5) compared with classical integration in Mnova 15.0.1 program. Colors correspond to regions marked in panel (A).

**Figure 4:**
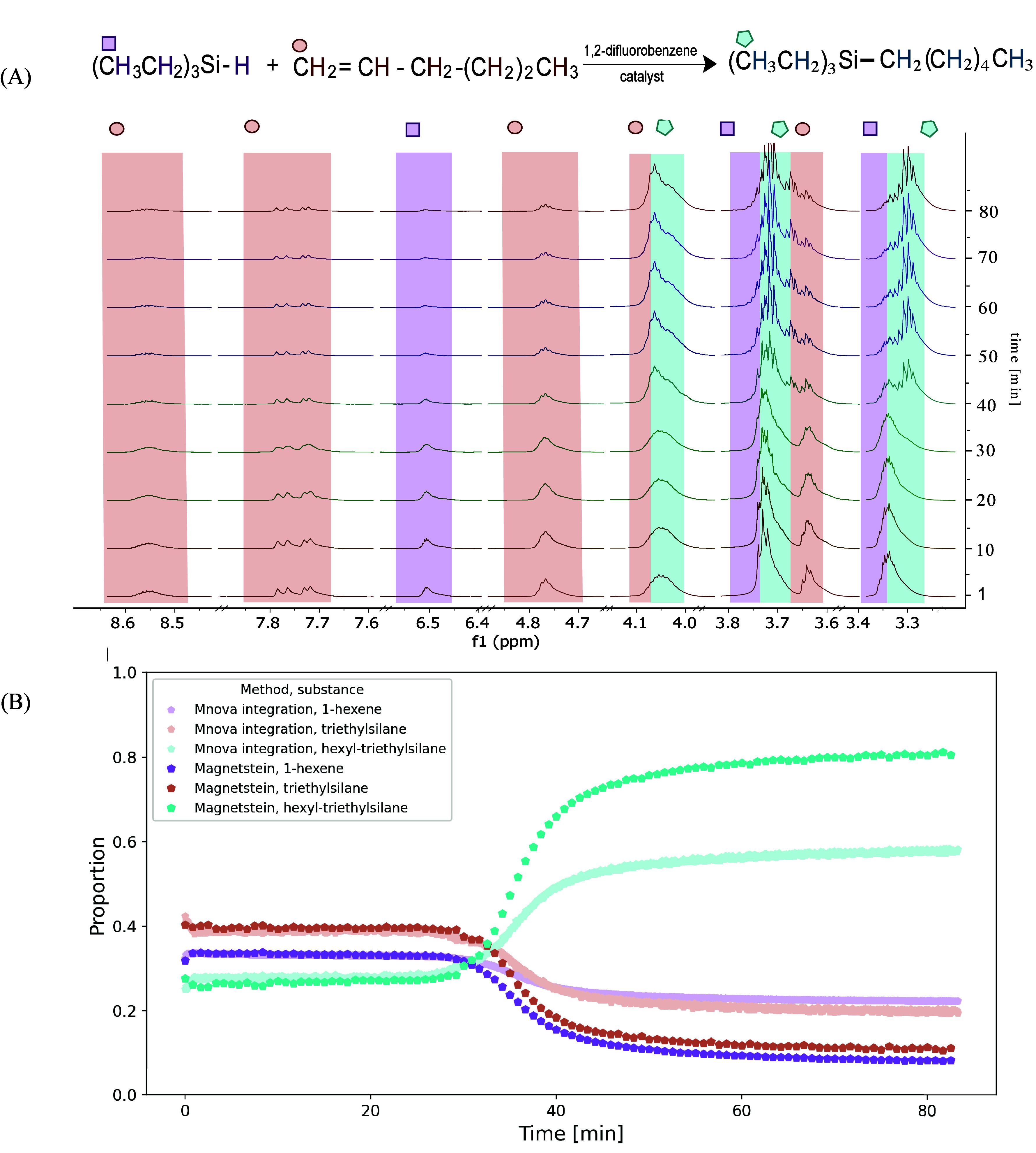
(A) Hydrosilylation reaction of 1-hexene and triethylsilane in 1,2-difluorobenzene, with 3 m% of a catalyst, with every 120th spectrum shown. The same colors and symbols mark the spectral peaks and the corresponding molecule in the reaction scheme. (B) The kinetics of hexene hydrosilylation. The results of Magnetstein processing (with κ_mixture_ = κ_components_ = 0.5) compared with classical integration in Mnova 15.0.1 program. Colors correspond to regions marked in panel (A).

In contrast, conventional integration methods treat each region independently, summing intensities without considering the influence of overlap. This independent summation introduces biases: product peak intensities can artificially contribute to substrate peak regions, and vice versa, leading to distorted results. Magnetstein’s ability to couple the regions and balance their interactions naturally mitigates these issues, providing more reliable kinetic curves even under severe spectral overlap.

Importantly, for the presented hydrosilyliation reactions, the model-based deconvolution could not solve the overlap problem, since it is impossible to assume the simple line shape model due to the shim distortion.

To further investigate the specific conditions under which Magnetstein gains advantage over traditional methods, we conducted an additional analysis on the simulated data set consisting of overlapping and shifting spectra. The results are described in the Supporting Information.

It may seem that the construction of the library is a bottleneck of the method, as for the spectra with huge overlaps, peaks shifting, or changing shape in time, an indication of proper areas corresponding to reagents can be infeasible. However, the proposed method is flexible and robust to such difficulties. Oftentimes, providing approximate regions where peaks from reagents are expected is sufficient for the algorithm to work properly. The approximate tolerance for peak shifting is a user-defined parameter (denoising penalty). The spectra in the library do not need to be complete, i.e. they can involve only well-separable, easy-to-interpret intervals cut out from the full chemical shift axis. Moreover, the library construction can be further simplified if the user is not interested in estimation for each individual reagent but rather in the product-to-substrate ratio. Note that this is sufficient to determine the reaction kinetics. In such a case, it is enough to cut out the regions corresponding to products and those corresponding to substrates as a whole without further splitting into individual compounds.

Concerning the spectra of the reaction mixture, one can either cut the spectra to the same regions as those in reagents or use the full spectra. The first approach is preferred, as in this case the algorithm works properly for a very wide range of parameters’ values, allowing a user to run the analysis with default settings. However, using full spectra of the reaction mixture and adjusting parameters accordingly is also possible. In the three reactions described above, the spectra of the reaction mixture were cut to regions. With such input data, Magnetstein proved to be extremely robust to the choice of parameters κ_mixture_ and κ_components_: the results were virtually the same independent of the setting, see Figure 5(A). As we mentioned, using the other approach is also possible but requires a more careful choice of the parameters values. To present the difference, we ran the algorithm for sucrose hydrolysis once again using the full spectra of the reaction mixture and a grid of values for κ_mixture_ and κ_components_. As presented in Figure 5(B), the results are correct for small values of the κ_mixture_ and κ_components_. This is consistent with the interpretation of the parameters: small values of the penalties allow for easy removal of excessive signal. Clearly, if we compare the full spectrum of the mixture with the cut-down spectra of reagents, the large amount of signal is redundant. Note that while using Magnetstein with such settings, one should rely rather on qualitative than quantitative results, as the considerable amount of spectral signal gets removed to the auxiliary points, perturbing the absolute values of proportions. Analogical analysis for the hydrosilylation reactions is presented in the Supporting Information.

**Figure 5:**
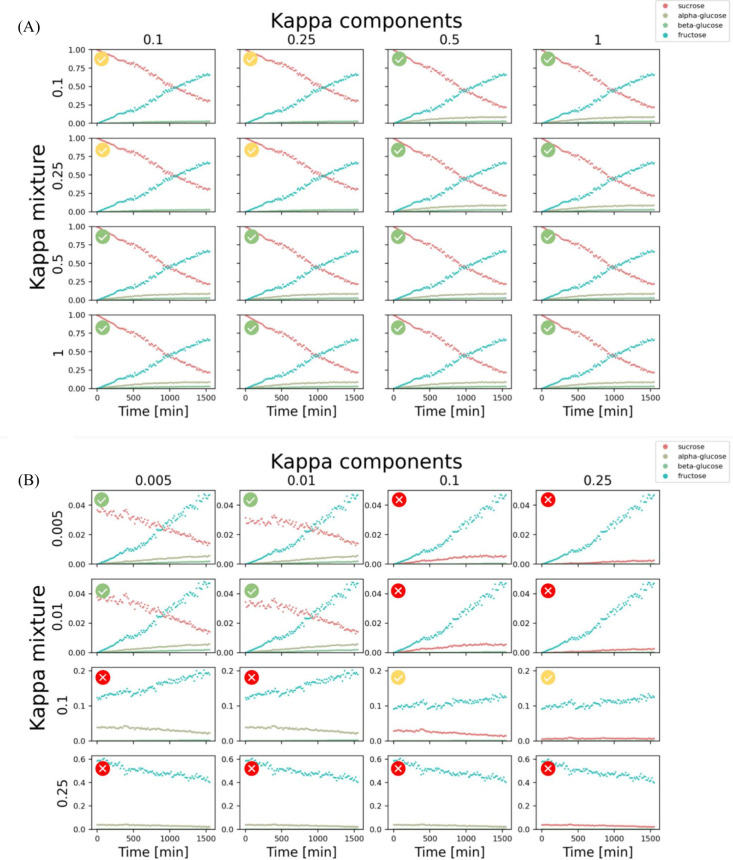
Comparison of Magnetstein results obtained for sucrose hydrolysis performed using a library of the cut (A) and the full (B) spectrum of the reaction mixture. The panels correspond to different settings of parameters κ_mixture_ and κ_components_. Colors correspond to regions marked in Figure 2(A).

## Conclusions

Tools based on optimal transport are widely applicable to signal-processing problems common to chemical analysis. Our work shows that these methods can also benefit the monitoring of chemical reactions by NMR spectroscopy. We present how the program Magnetstein, based on the optimal transport Wasserstein metric, allows for efficient determination of the reaction kinetics. Contrary to conventional approaches, the method works well even for spectra with significant peak overlap and nonstandard lineshapes caused, for example, by magnetic field inhomogeneity. The method has only two parameters, which can be set to constant values for many data sets.

## Supplementary Material



## Data Availability

Python package with algorithm implementation is available at https://github.com/BDomzal/magnetstein. The code for reproducing experiments (involving preprocessing, estimation, and visualizations) is available at https://github.com/BDomzal/magnetstein_x_chemical_reactions. All data sets are available at https://zenodo.org/records/14814657.

## Notes

The Magnetstein algorithm; computational optimization using information about time; results for incomplete library; library construction; guidebook for the users; investigating the impact of peak overlap and shift; results for different values of parameters in case of cut and full reaction mixture spectra (PDF)
