# Supplementary material for: NMR Reaction Monitoring Robust to Spectral Distortions
Source: Anal Chem. 2025 Jul 16;97(29):15633–41. doi: 10.1021/acs.analchem.5c00800 (PMC12311900; doi:10.1021/acs.analchem.5c00800)
Supplement: Supplementary file 1 [file ac5c00800_si_001.pdf]

Supporting Information

NMR reaction monitoring robust to spectral distortions

Barbara Domżał[1], Magdalena Grochowska-Tatarczak[2], Przemysław Malinowski[2], Błażej Miasojedow[1], Krzysztof Kazimierczuk*[2], and Anna Gambin*[1]

[1]Faculty of Mathematics, Informatics and Mechanics, University of Warsaw, Banacha 2, Warsaw, 02-097, Poland.
[2]Centre of New Technologies, University of Warsaw, Banacha 2C, Warsaw, 02-097, Poland.

Contents

1 The Magnetstein algorithm S3
2 Computational optimization using information about time S4
3 Results for incomplete library S5
3.1 Sucrose hydrolysis S5
4 Library construction S6
5 Guidebook for the users S7
5.1 When to use Magnetstein instead of traditional integration methods S7
5.2 What to use as an input S7
5.3 How to interpret and set the values of the parameters S8
5.4 How to interpret the output S8
5.5 Reproducibility S8
5.6 Where to read more S8
6 Investigating the impact of peak overlap and shift S8
7 Results for different values of parameters in case of cut reaction mixture spectra S9
7.1 Pentene hydrosilylation S9
7.2 Hexene hydrosilylation S10
8 Results for different values of parameters in case of full reaction mixture spectra S11
8.1 Pentene hydrosilylation S11
8.2 Hexene hydrosilylation S12

References **S14**

1 The Magnetstein algorithm

To describe the theory behind the Magnetstein algorithm, first we need to introduce a non-standard way of representing an NMR spectrum. Conventionally, spectra are represented as vectors, where the coordinates correspond to signal intensities in consecutive points of the chemical shift axis. Here, in contrast, we propose to view a spectrum as a probability distribution. Note that introducing this representation is a purely technical operation performed to be able to use the Wasserstein distance later on. We do not suggest any probabilistic interpretation of spectra. In practice, viewing spectra as probability distributions means storing the data as a set of pairs, where the first element corresponds to chemical shift and the second one corresponds to intensity. The intensities need to be normalized so that they sum up to 1. Note that this approach does not require the common measurement points for the analyzed spectra, and we can still meaningfully compare spectra with different chemical shift axes.

Now, we can introduce the notion of the Wasserstein metric [2], denoted as W, which is a distance function defined between the two probability distributions representing spectra, denoted as μ and ν. Intuitively, the Wasserstein distance is the lowest possible cost required to transform distribution μ into distribution ν by transporting probability mass along the horizontal axis. The cost of each move is equal to the amount of the transported mass (spectral intensity) multiplied by the distance on which the mass was transported. A total cost is the sum of the costs of all the individual moves.

The special properties of the Wasserstein distance make it a particularly well-suited tool for the analysis of spectroscopic data. Firstly, this metric is robust to measurement inaccuracies and shifts along the horizontal axis, as transporting even considerable amounts of mass over small distances is not costly. In practice, it means that for the two spectra that share the intensities of peaks but slightly differ in their positions or lineshapes, the Wasserstein distance will be small. It can be calculated for spectra with distorted, irregular lineshapes resulting from non-homogeneous magnetic fields or peak overlap. Finally, as already mentioned, thanks to representing spectra as probability distributions instead of vectors, any two spectra can be compared this way, regardless of their resolution and spectral window.

Although computing the Wasserstein distance is an apparently complex problem, it has been proven [2] that in the one-dimensional case, it can be easily and efficiently solved. Let M and N be the cumulative distribution functions of μ and ν, respectively. For the convenience of notation, let us introduce a set $S = \{s_1, s_2, \ldots, s_n\}$ being a union of all the points on chemical shift axes of μ and ν. Without loss of generality, we can assume that s_i are sorted so that $s_i < s_{i+1}$. We will denote the intensities of spectra in consecutive points as $\mu(s_i)$ and $\nu(s_i)$ for $i = 1, \ldots, n$. For those s_i that were not originally present in spectrum μ, we will simply set $\mu(s_i) = 0$ and, similarly, for those that were not present in ν, we will set $\nu(s_i) = 0$. Then, the formula for efficient computation of the Wasserstein distance is as follows:

$$W(\mu, \nu) = \sum_{i=1}^{n-1} |M(s_i) - N(s_i)| \cdot (s_{i+1} - s_i).$$

Now that we know how to compute the distance between two spectra, let us proceed to the description of the Magnetstein algorithm. Suppose that we want to estimate $p_{j,t}$ for $j = 1, ..., k$ and fixed moment t, and that the spectrum μ_t of reaction mixture and spectra $\nu_1, \nu_2, \ldots, \nu_k$, constituting a library (independent of t), are all available. Let $p_t = (p_{1,t}, p_{2,t}, \ldots, p_{k,t})$ be the unknown proportions of spectra $\nu_1, \nu_2, \ldots, \nu_k$ in moment t, and let us denote the linear combination of library's spectra added in proportions p_t as $\nu_{p_t} = p_{1,t}\nu_1 + p_{2,t}\nu_2 + \cdots + p_{k,t}\nu_k$. The problem of finding the unknown proportions p_t can be formulated as regression with the Wasserstein distance:

$$\min_{p_t} W(\mu_t, \nu_{p_t})$$

for all $t = 1, 2, \ldots, T$. We further developed this approach [1], by assuming that some parts of spectra μ_t and ν_{p_t} can be excessive or missing, i.e. both μ_t and ν_{p_t} can contain noise and signals from contamination but also can lack signals that are expected. This assumption is modelled by adding the auxiliary points ω (that represents signal present in μ_t, but missing in ν_{p_t}) and ξ (that represents signal present in ν_{p_t}, but missing in μ_t). The proportion of signal removed to those points is equal to $p_{0,t}$ and $p'_{0,t}$ for points ω and ξ, respectively. The variables $p_{0,t}$ and $p'_{0,t}$ can be treated as the measure of quality for the input spectra of reaction mixture and of the library, respectively: the higher their values, the more signal is removed and,

therefore, the worse the quality of the input data. To properly define the Wasserstein distance for this new augmented space, we need to define the cost of signal transport to auxiliary points ω and ξ. We assume that transport from any point to ω costs $\kappa_{mixture}$ and transport from any point to ξ costs $\kappa_{components}$. These are so-called *denoising penalties* that are user-defined parameters of the Magnetstein algorithm and can be interpreted as soft tolerance thresholds for shifting of the signal along the horizontal axis for the spectrum μ_t and for the library $\nu_1, \nu_2, \ldots, \nu_k$, respectively. After including these new assumptions, the final formulation of the problem is as follows:

$$\min_{p_t, p'_{0,t}} W_\kappa \Big((1 - p'_{0,t})\mu_t + p'_{0,t}\xi, \nu_{p_t} + p_{0,t}\omega \Big), \tag{1}$$

where

$$p_{0,t} = 1 - p_{1,t} - p_{2,t} - \cdots - p_{k,t},$$
$$0 \le p'_{0,t}, p_{0,t}, p_{1,t}, \ldots, p_{k,t} \le 1$$

and W_κ denotes the Wasserstein distance with possibility of transport to ω with cost κ_{mixture} and to ξ with cost $\kappa_{\text{components}}$. The optimal values $p_{1,t}, p_{2,t}, \ldots, p_{k,t}$ obtained after solving (1) are the sought proportions of $\nu_1, \nu_2, \ldots, \nu_k$, while the value of the Wasserstein distance can be qualitatively interpreted as a measure of lineshape deformations and peak shifts between the input spectra of the reaction mixture and the library.

The detailed procedure for solving (1) is implemented in the Magnetstein Python package [1]. This optimization problem boils down to a linear program. As mentioned in the main text, the program can be solved separately and independently for every fixed moment t. However, at this point, we make use of the knowledge that spectra μ_{t-1} and μ_t, being close in time, are expected to be similar to one another (see next section for details).

2 Computational optimization using information about time

As stated in the previous section, the task of estimating reagents' proportions in the fixed moment t can be formulated as follows:

$$\min_{p_t, p'_{0,t}} W_\kappa \Big((1 - p'_{0,t})\mu_t + p'_{0,t}\xi, \nu_{p_t} + p_{0,t}\omega \Big), \tag{2}$$

where

$$p_{0,t} = 1 - p_{1,t} - p_{2,t} - \cdots - p_{k,t},$$
$$0 \le p'_{0,t}, p_{0,t}, p_{1,t}, \ldots, p_{k,t} \le 1$$

and W_κ denotes the Wasserstein distance with possibility of transport to ω with cost κ_{mixture} and to ξ with cost $\kappa_{\text{components}}$.

For simplicity of notation, let us denote κ_{mixture} (penalty for removing noise signal from spectrum μ_t) as κ and $\kappa_{\text{components}}$ (penalty for removing signal from spectrum ν_{p_t}) as κ'.

Let us recall the theoretical result proved by Domżał et al.[1]:

Theorem 1. *Let $S = \{s_1, s_2, ..., s_n\}$ be an ordered list of all distinct ppm values present in all the spectra. Problem of finding*

$$p^* = \arg\min_p W_{\rho_2}((1 - p'_0)\mu + p'_0\xi, \nu_p + p_0\omega)$$

with distance function

$$\rho_2(x, y) = \begin{cases} |x - y| & \text{if } x, y \in \mathbb{R}, \\ \kappa & \text{if } x \in \mathbb{R}, y = \omega, \\ \kappa' & \text{if } x = \xi, y \in \mathbb{R}, \\ \kappa + \kappa' & \text{if } x = \xi, y = \omega. \end{cases}$$

is equivalent to a linear program

$$
\begin{aligned}
\text{maximize} \quad & V^T \boldsymbol{z} \\
\text{subject to} \quad & (V')^T \boldsymbol{z} \leq \kappa', \\
& W^T \boldsymbol{z} \leq -\kappa, \\
& z_i - z_n \leq 0 \quad \textit{for } i = 1, 2, ..., n-1, \\
& -z_i - z_{n+1} \leq 0 \quad \textit{for } i = 1, 2, ..., n-1, \\
& z_i - z_{i+1} \leq l_i \quad \textit{for } i = 1, 2, ..., n-2, \\
& z_{n-1} \leq l_{n-1}, \\
& z_i - z_{i+1} \geq -l_i \quad \textit{for } i = 1, 2, ..., n-2, \\
& z_{n-1} \geq -l_{n-1}, \\
& z_n, z_{n+1}, z_{n+2}, z_{n+3} \geq 0, \\
& \boldsymbol{z} \in \mathbb{R}^{n+3},
\end{aligned}
$$

where

$$
V := \begin{bmatrix} \mu(s_1) & \mu(s_2) & ... & \mu(s_{n-1}) & -1 & 0 & 0 & -1 \end{bmatrix}^T,
$$

$$
V' := \begin{bmatrix} \mu(s_1) & \mu(s_2) & ... & \mu(s_{n-1}) & 0 & 1 & -1 & 0 \end{bmatrix}^T,
$$

$$
W := \begin{bmatrix} \nu_1(s_1) & \nu_2(s_1) & ... & \nu_k(s_1) \\ \nu_1(s_2) & \nu_2(s_2) & ... & \nu_k(s_2) \\ & & ... & \\ \nu_1(s_{n-1}) & \nu_2(s_{n-1}) & ... & \nu_k(s_{n-1}) \\ -1 & -1 & ... & -1 \\ 0 & 0 & ... & 0 \\ 1 & 1 & ... & 1 \\ -1 & -1 & ... & -1 \end{bmatrix},
$$

$$
l_i := s_{i+1} - s_i.
$$

Clearly, by setting $\mu := \mu_t$, $\nu_p := \nu_{p_t}$, $p := p_t$ and $p'_0 := p'_{0,t}$ for every fixed $t = 1, \ldots, T$, we can reformulate the problem (2) as a linear program using the above theorem. In such a way we obtain a set of linear programs indexed by t.

The linear programs are solved consecutively one after another by the simplex algorithm. The algorithm can be *warm-started*, i.e. we can provide an initial guess for the solution, to prevent the algorithm from randomly starting the search in some point located far away from the optimal one. Here we propose the values from the previous moment $t-1$ as the initial guess: having solved the problem (2) for $t-1$, we pass the resulting values as a warm-start for problem (2) at t. This modification of the algorithm is implemented in the Magnetstein Python package.

3 Results for incomplete library

3.1 Sucrose hydrolysis

To illustrate that Magnetstein can work with an incomplete library, we compared the results from five different runs of the algorithm for sucrose hydrolysis data (see Figure S1). The first run was the standard one: all the components were added to the library. The four other runs corresponded to situations when one of the components was excluded from the library. Each of the four graphs corresponding to excluded components contains an additional grey curve – a proportion of the removed signal.

What turns out, is that in the absence of a particular component in the library, the removed signal curve behaves exactly like the curve corresponding to this missing component. We observe the exact same kinetics in each of the five graphs, but only the colors are changed to distinguish the proportion of the removed signal.

Note that to obtain the results shown in the figures below, we needed to set the parameters κ_{mixture}, $\kappa_{\text{components}}$ to non-default values (0.1, 1.0, respectively). This is expected, as those parameters let us express our confidence in the purity of the mixture spectrum and in the reliability of the library. In this case, we decreased κ_{mixture} to 0.1 to express the suspicion of the presence of the contaminants from outside the library. On the other hand, $\kappa_{\text{components}}$ was increased to 1.0, reflecting our emphasis on detecting every species included in the library.

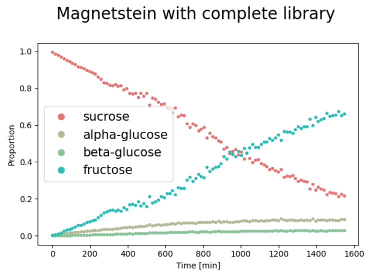

(a)

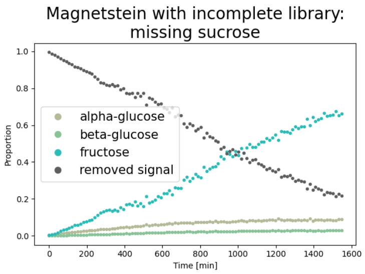

(b)

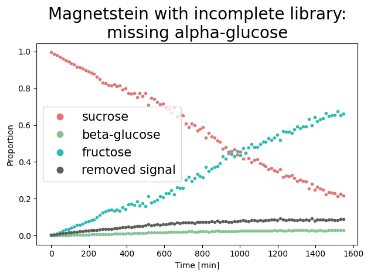

(c)

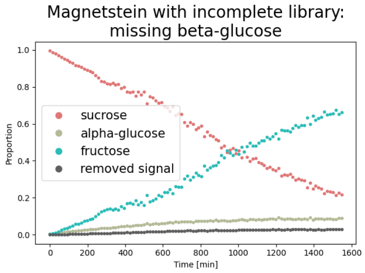

(d)

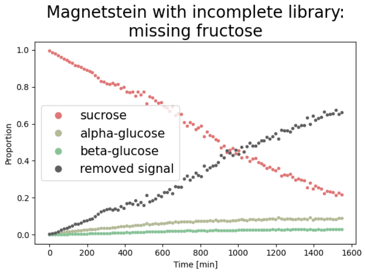

(e)

Figure S 1: The kinetics of sucrose hydrolysis. The results of Magnetstein processing (with $\kappa_{\text{mixture}} = 0.1$, $\kappa_{\text{components}} = 1.0$) with complete (1a) or incomplete (1b-1e) library as input. For cases (1b-1e), the graph of removed signal can serve as a reconstruction of the missing reagent's kinetics.

4 Library construction

Experiment	Reagent	Time point number	Regions
Sucrose hydrolysis	sucrose	1001	$(5.39173, 5.44305)$
	α-glucose	1001	$(5.2178, 5.26134)$
	β-glucose	1001	$(4.62026, 4.67207)$
	fructose	1000	$(3.97917, 4.01542)$
Pentene hydrosilylation	pentene	920	$(5.3, 5.6)$
	triethylsilane	920	$(3.6, 3.95)$
	2-pentyl-1-triethylsilane	920	$(1.16, 1.36)$
Hexene hydrosilylation	hexene	250	$(3.599926, 3.679897)$, $(4.079348, 4.129027)$, $(4.668059, 4.857709)$, $(7.616258, 7.838666)$, $(8.440374, 8.647265)$
	triethylsilane	250	$(3.343730, 3.406997)$, $(3.756886, 3.832615)$, $(6.422073, 6.564104)$
	hexyl-triethylsilane	250	$(3.256018, 3.343730)$, $(3.681636, 3.756886)$, $(3.994139, 4.078496)$

Table 1: Information about construction of the library. The elements of the library are cut from one of the spectra of reaction mixture. The column 'Time point number' indicates which spectrum of reaction mixture is used for this purpose. The column 'Regions' stores the chemical shift intervals that are cut from the spectrum of reaction mixture to represent individual reagents in the library.

5 Guidebook for the users

This section serves as a short guidebook on the usage of Magnetstein for the analysis of chemical reactions.

5.1 When to use Magnetstein instead of traditional integration methods

The method should be used when:

- peaks move throughout the reaction,
- peaks from different reagents overlap,
- lineshapes are distorted,
- there are contaminations and/or noise.

5.2 What to use as an input

The input should consist of the two crucial parts:

- mixture, i.e., a series of NMR spectra of the reaction mixture measured in consecutive moments,
- library, i.e., a set of spectra of individual reagents expected to be present in the mixture (no need for a series here, just a single spectrum for each reagent).

5.3 How to interpret and set the values of the parameters

The user needs to define the values of two parameters: $\kappa_{mixture}$ and $\kappa_{components}$. These are so-called *denoising penalties*, that can be interpreted as soft tolerance thresholds for shifting of the signal along the horizontal axis for the spectrum of the reaction mixture and for the spectra in the library, respectively.

Another interpretation is viewing $\kappa_{mixture}$ as a certain measure of reliability of the mixture's spectrum. The higher its value, the less likely the algorithm is to remove noise from the spectrum. Similarly, $\kappa_{components}$ reflects our confidence in the purity of the spectra in the library.

If you are unsure how to set the parameters, we recommend using the default values, i.e., $\kappa_{mixture} = 0.25$ and $\kappa_{components} = 0.22$. We checked experimentally that such settings produced accurate results for many datasets.

5.4 How to interpret the output

The main output of the algorithm is a series of vectors indexed by time:

$$p_t = \Big(p_{1,t}, p_{2,t}, \ldots, p_{k,t}\Big),$$

where $p_{i,t}$ is the *proportion* of the i-th reagent in the reaction mixture. By *proportions* here we mean the relative amounts of reagents. Note that $p_{1,t} + p_{2,t} + \cdots + p_{k,t}$ does not necessarily equal to 1 due to the presence of noise and contamination. The quantity $p_{0,t} := 1 - p_{1,t} - p_{2,t} - \cdots - p_{k,t}$ is Magnetstein's estimation of the relative amount of the signal coming from the contamination in the reaction mixture's spectrum. Similarly, the quantity $p'_{0,t}$, also returned by the algorithm, is Magnestein's estimation of the relative amount of the signal coming from the contamination in the library.

5.5 Reproducibility

The output of the program depends solely on two factors:

- input data, i.e., a series of spectra of the reaction mixture indexed by time, and the library,
- parameter settings, i.e. chosen values of $\kappa_{mixture}$ and $\kappa_{components}$.

If these are provided, the output can be reproduced.

5.6 Where to read more

You can find more instructions at `https://github.com/BDomzal/magnetstein_x_chemical_reactions` and `https://github.com/BDomzal/magnetstein`.

6 Investigating the impact of peak overlap and shift

The results obtained for hexene hydrosilylation suggest that Magnetstein gains an advantage over traditional integration in case of overlapping spectra. To further investigate this matter, we performed an additional computational experiment on simulated data.

The simulated mixture's spectrum consisted of two Lorentzian peaks (with identical shapes but different locations). The location of the first peak was fixed, while that of the second peak was changed to obtain different amounts of overlap between the two spectra. The overlap proportion ranged from 0.02 of the total area under the peak to 1.0 (corresponding to one peak effectively present in the mixture's spectrum). In this way, we obtained 99 mixtures.

The library was constructed separately and automatically for each mixture, simply by cutting the spectrum of the mixture to two parts halfway between the maxima of the two peaks. The left cut part and the right cut part became the two components in the library.

To investigate the impact of peak shifting on the results, the mixture spectra were further processed after the library construction: that is, each of the 99 mixture spectra was modified by shifting the entire spectrum

to the right. The value of the shift ranged from 0.001 to 0.501 ppm. This way, we obtained a total of $99 \cdot 50$ mixture spectra, corresponding to 99 values of the overlap proportion and 50 values of the shift.

Each of the mixtures, together with the library constructed from it, was then analysed by Magnetstein. Both parameters $\kappa_{\mathrm{mixture}}$ and $\kappa_{\mathrm{components}}$ were set to the same value, equal to the shift of the given mixture.

The results are presented in Figure S2. As we can observe, Magnetstein is robust both to the overlapping and to the shifting of peaks if these two problems do not occur at the same time. Clearly, the values of absolute error remain small for a wide range of shift values if the overlap percentage between the two peaks is small. Similarly, if the peak positions are stable, the large overlap does not perturb the results. The mixed situations remain a challenge for the algorithm.

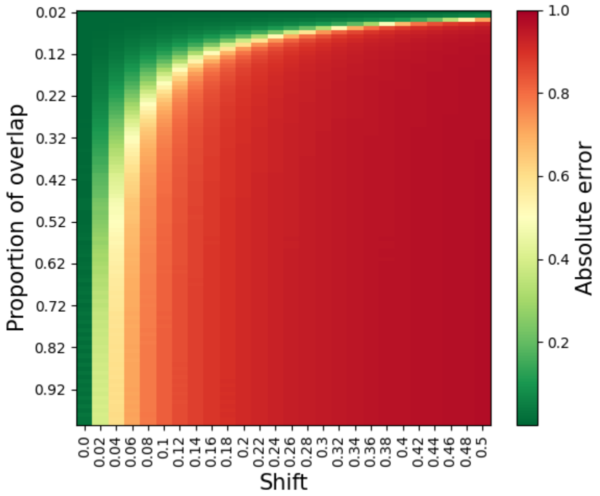

Figure S 2: Absolute error of Magnetstein's estimation for 99 simulated mixture spectra with two peaks, each from one component. The overlap and shift between the library and mixture were varied.

7 Results for different values of parameters in case of cut reaction mixture spectra

7.1 Pentene hydrosilylation

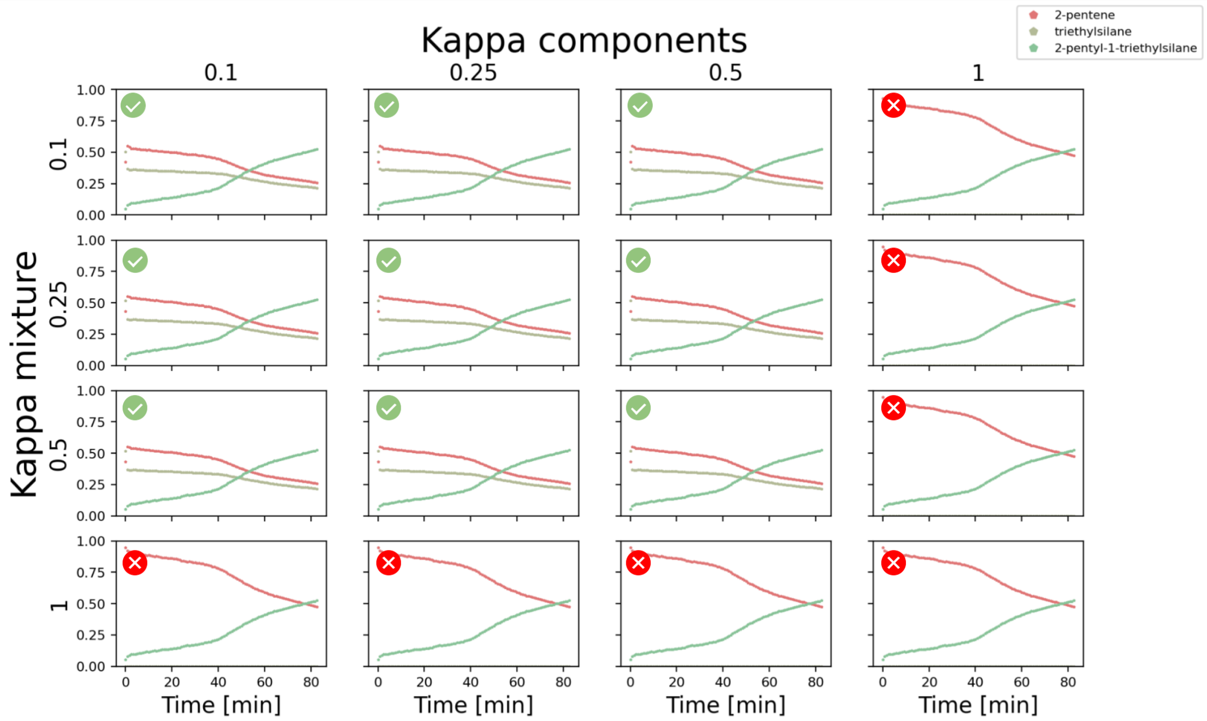

Figure S 3: Comparison of results obtained for pentene hydrosilylation from Magnetstein ran on the cut spectrum of reaction mixture for a grid of parameters κ_{mixture} and $\kappa_{\text{components}}$. Colors correspond to regions marked in Figure 3.

7.2 Hexene hydrosilylation

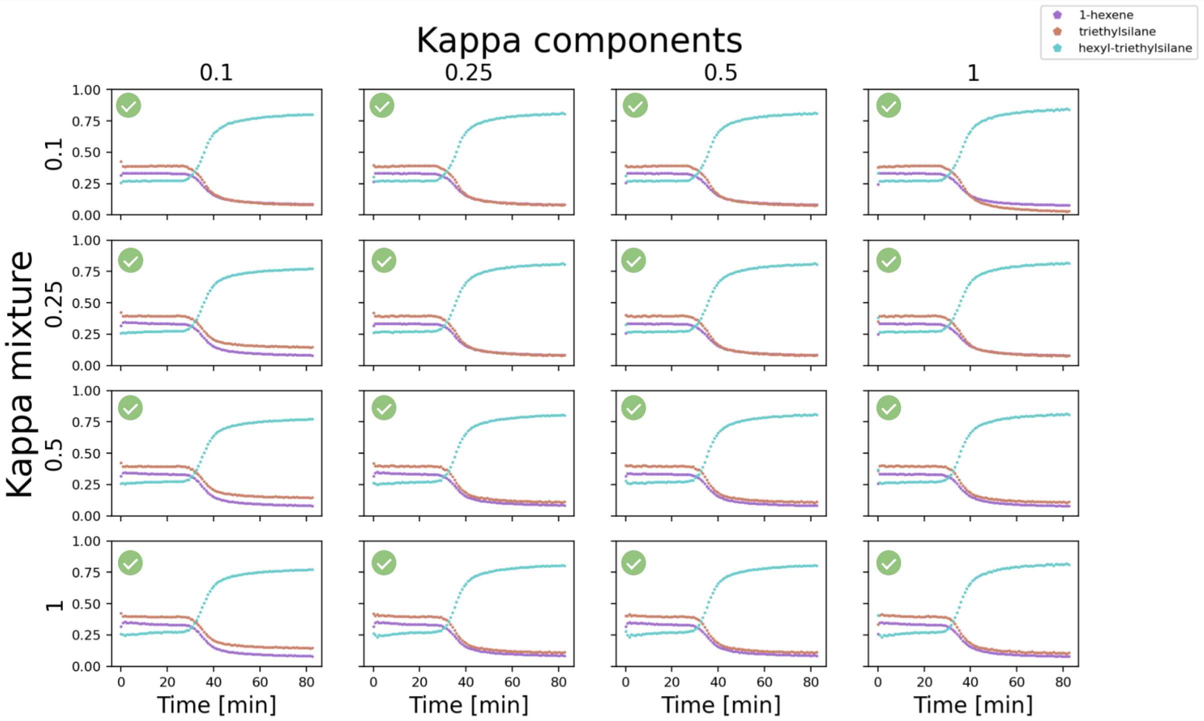

Figure S 4: Comparison of results obtained for hexene hydrosilylation from Magnetstein ran on the cut spectrum of reaction mixture for a grid of parameters κ_{mixture} and $\kappa_{\text{components}}$. Colors correspond to regions marked in Figure 4.

8 Results for different values of parameters in case of full reaction mixture spectra

8.1 Pentene hydrosilylation

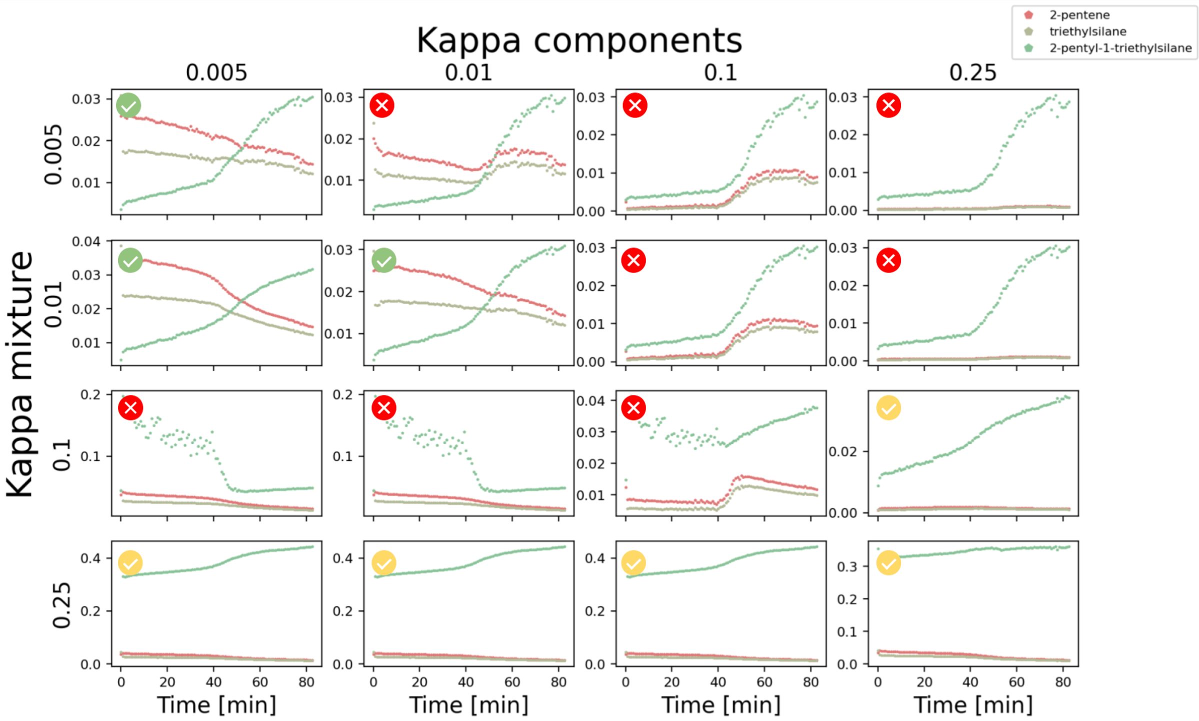

Figure S 5: Comparison of results obtained for pentene hydrosilylation from Magnetstein ran on the full spectrum of reaction mixture for a grid of parameters $\kappa_{\mathrm{mixture}}$ and $\kappa_{\mathrm{components}}$. Colors correspond to regions marked in Figure 3.

8.2 Hexene hydrosilylation

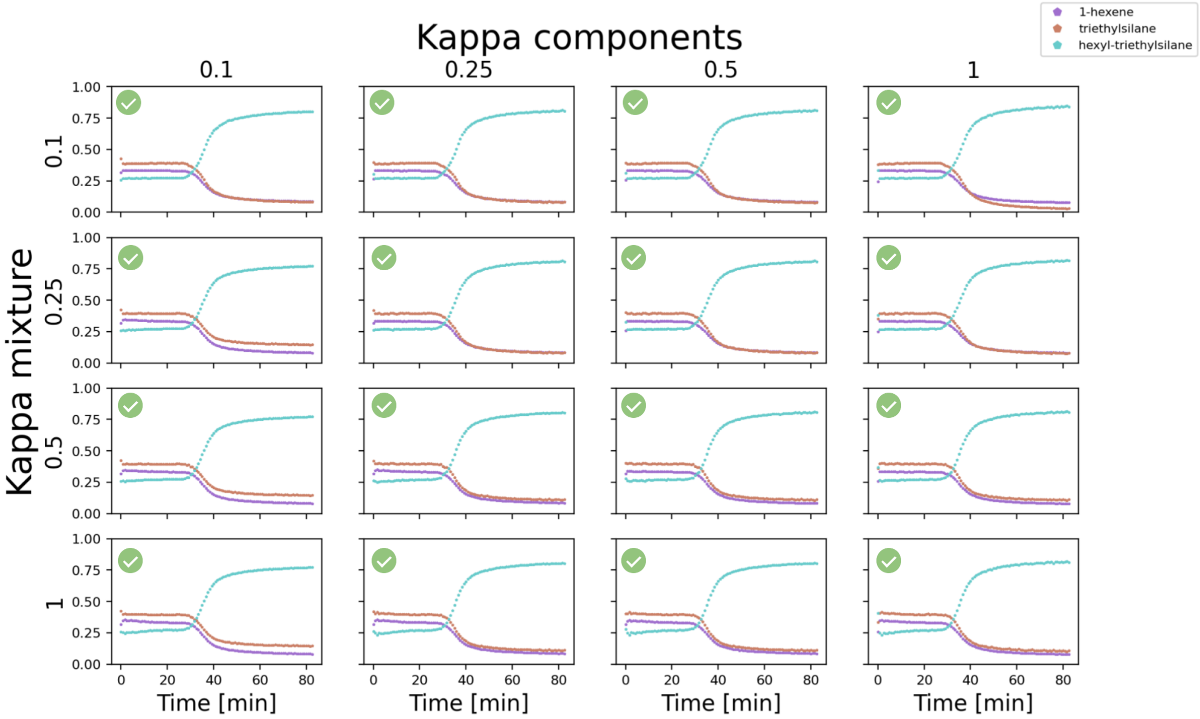

Figure S 6: Comparison of results obtained for hexene hydrosilylation from Magnetstein ran on the full spectrum of reaction mixture for a grid of parameters κ_{mixture} and $\kappa_{\text{components}}$. Colors correspond to regions marked in Figure 4.

Bibliography

[1] Domżał, B.; Nawrocka, E. K.; Gołowicz, D.; Ciach, M. A.; Miasojedow, B.; Kazimierczuk, K.; Gambin, A. Magnetstein: An Open-Source Tool for Quantitative NMR Mixture Analysis Robust to Low Resolution, Distorted Lineshapes and Peak Shifts. *Anal. Chem.* **2023**, *96*, 188-196.

[2] Santambrogio, F. Optimal transport for applied mathematicians. *Birkäuser, NY* **2015**, 177-183.
